# Clean your own house first: integrating sustainability into microbiology labs

**DOI:** 10.1093/femsec/fiae084

**Published:** 2024-05-30

**Authors:** Priscilla Carrillo-Barragan

**Affiliations:** University of Basel, Man-Society-Environment Group, Department of Environmental Science, Basel, Vesalgasse 1 CH-4051, Switzerland

**Keywords:** environmental, future, impact, laboratory, microbiology, plastic pollution, sustainability, sustainable

## Abstract

Microbiology laboratories are pivotal hubs for exploring the potential of microorganisms and addressing global challenges. Particularly, Environmental Microbiology facilities hold substantial influence in advancing knowledge and capabilities crucial for achieving the United Nations Sustainable Development Goals. This raises the imperative of integrating sustainable practices to mitigate the environmental impact of research activities and foster a culture of responsibility. Such an approach not only aligns with global sustainability objectives but also catalyses innovative, eco-conscious methodologies in scientific research aimed at tackling pressing environmental issues. Concerns regarding the environmental footprint of laboratory practices have stimulated innovative improvements within the scientific community, ranging from resource-efficient initiatives to the management of essential commodities like water and energy. This perspective discusses specific areas where microbiology laboratories can enhance their sustainability efforts, drawing on reports and case studies of pioneering groups. Additionally, it explores potential collaborators to support these endeavours and emphasises the pivotal role of early career researchers in driving this transition. By initiating discussions and sparking curiosity within the environmental microbial community, this commentary seeks to propel the microbial ecology field toward a greener future, starting from within the laboratory environment.

## Teaser

Conversation on integrating sustainability in Microbiology labs to minimise environmental impact, aligning with the goal of addressing global environmental challenges through responsible research practices.

Microbiology laboratories play a pivotal role in understanding and harnessing the power of microorganisms. For instance, microbiology facilities can significantly advance our knowledge and capabilities to address the United Nations Sustainable Development Goals (Fagunwa and Olanbiwoninu 2020). This potential then warrants the question of whether it is equally important to integrate sustainable practices to minimise the environmental impact of our research and promote a culture of responsibility. This approach would not only align with global sustainability goals but also pave the way for innovative, eco-conscious methodologies in scientific research consistent with the vision of contributing to the solutions of the environmental challenges the world faces.

Indeed, concern regarding green practices in lab facilities has already fuelled innovative improvements and schemes within the scientific community (Madhusoodanan 2020, Akinsemolu 2023). Initiatives range from efficient resource use, including plastic use assessments (Alves et al. 2021) to water and energy management (Mathew et al. 2004, Stephanie Tanner 2005), all essential commodities in daily microbiology laboratories operations.

Here, we discuss specific areas where microbiology laboratories can advance the sustainability of their operations, referring to reports and case studies of groups already moving towards a greener direction. Moreover, potential collaborators that can support these changes, such as research institutions and learned societies are discussed. In addition, the role of early career researchers (ECR), team leaders, and other laboratory members in making this transition possible is highlighted. By doing so, this opinion piece aims to spark curiosity and kindle conversations among the FEMS microbiology community about how the field can progress sustainably from the inside out.

## Areas for assessment and improvement in laboratory practices

### Energy efficiency

A critical step towards sustainability is reducing energy consumption. Microbiology labs, with their array of equipment like fridges, freezers, biosafety cabinets, autoclaves, incubators, thermo cyclers, and centrifuges, to name a few, are typically energy intensive. Simple measures like turning off equipment when not in use, defrosting freezers, avoiding blocking air vents in biosafety cabinets, using LED lighting, and maintaining equipment for optimal performance can make a considerable difference.

Notably, the National Renewable Energy Laboratory, the Lawrence Berkeley National Laboratory, and the International Institute for Sustainable Laboratories have produced a report on assessing baseline energy performance for laboratories (Mathew et al. 2004). They also presented The Environmental Performance Criteria (EPC)—a point-based rating system designed to score overall environmental performance for laboratories (Mathew et al. 2004). Updated versions for both, the Laboratory Benchmarking energy use (I2SL 2005a) and EPC (I2SL 2023) tools are available online and are free to use. These institutes have further developed guidelines and a Smart Labs Toolkit to help increase environmental sustainability in laboratories (I2SL 2005b), currently used by numerous academic, public, and industrial laboratories.

Additionally, agreements with manufacturers and suppliers could facilitate upgrading to energy-efficient models, significantly cutting down on power usage. Furthermore, questioning our long-standing practices could also lead to energy savings. For example, a discussion on Twitter raised the question of whether setting a freezer at –80°C is necessary. Could increasing the temperature to –70°C also suffice? Leak and collaborators explored this question, finding that operating an energy-efficient freezer at –70°C, if necessary, with empty boxes to buffer against temperature changes, can reduce energy use by about 36% compared to running the same model at –80°C (Leak et al. 2023). Further, the Freezer Challenge organisation has made available scientific evidence that –70°C is a safe storage temperature for various sample types (Freezer Challenge 2016), including fungal isolates (Espinel-Ingroff et al. 2004) and proteins (Beekhof et al. 2012). Indeed, together with the University of Colorado Boulder, the Freezer Challenge has created a database where life scientists across the world share the type of samples stored at –70°C, their applications, and safe storage tests conducted. These initiatives are open invitations for more microbiology labs to participate.

Granting social media might not seem like the ideal environment for academic discussion, but it can provide a space for open conversations about long-standing beliefs, leading to useful knowledge.

Economic benefits further support sustainability practices. This has been proven by the Harvard University's Green Labs Program, which by encouraging practices such as shutting fume hoods when not in use, has saved approximately $250 000 USD annually in energy costs, reducing the carbon footprint and improving laboratory safety and efficiency by maintaining optimal airflow (Quentin 2017).

### Water conservation

Water is a critical resource in microbiology labs, used for everything from washing glassware to preparing solutions and culture media. In settings where autoclaves and dishwashers are the major water consuming pieces of equipment, running these only when completely full and efficiently loaded would greatly improve the efficient use of this resource. Coordinating with other laboratories could help maximise the use of each cycle. Equally, assessing the type of water necessary for each application can reduce water consumption. For instance, considering that producing 1 L of deionised water requires 3 L of tap water (Leak et al. 2023), should prompt the evaluation of whether a given activity needs deionised water. Other advisable changes include using foot-pedals in lab sinks to facilitate turning water on and off and recirculating water systems for cooling laboratory equipment (Royal Society of Chemistry 2022).

The University of Bristol has implemented various water-saving measures in their laboratories, including in those dedicated to medical microbiology and infectious disease. For example, their Green Labs scheme, focused on optimising water usage among other sustainability goals. Measures include installing low-flow aerators on lab sinks, maintaining reverse-osmosis systems efficiently, and actively identifying and fixing leaks. This proactive maintenance has prevented water loss and potential damage to lab equipment and facilities, ensuring smoother laboratory operations by reducing equipment downtime​, ultimately allowing the University to allocate more resources to research activities (The University of Bristol 2017, 2022).

### Chemical use and disposal

Chemicals are indispensable in microbiology labs, to the extent, that we can all think of containers with reagents from years past, often with the same chemical being ordered over and over again without any given jar being actually empty. Thus, by carefully managing inventory and avoiding overordering, labs can prevent waste from expired or unused chemicals. Also, the choice and disposal of chemicals impacts sustainability (Royal Society of Chemistry 2022). Eliminating or exchanging our reagents for less or non-hazardous compounds (EFLM 2022) and centralising their use and disposal at the department or institutional level can help to improve research reproducibility and sustainability practices (Meyn et al. 2022) while minimising the type and amount of chemical waste produced by individual laboratories.

### Waste management

Microbiology laboratories generate a variety of wastes, with single-use plastics likely constituting the largest proportion. The complexity of recycling plastics due to biological contaminants and mixed waste makes it challenging.

Although plastics used to manufacture laboratory consumables are technically recyclable (e.g. polypropylene), the presence of biological contaminants, the diversity of plastic types, and the mixed nature of waste make recycling a complex and often unfeasible option. Furthermore, almost all single-use plastic waste in microbiology labs is routinely collected in biohazard containers or bags for disposal and incinerated (Tan et al. 2022). However, a significant amount of the plastic waste discarded by research laboratories is not biohazardous. Indeed, a report analysing the recycling potential of plastic waste produced by clinical facilities in the United States found that plastic waste can significantly be reduced by appropriate segregation of waste (Lee et al. 2002).

In fact, some alternative strategies are already being implemented. For instance, the University of York waste audit and plastics decontamination station development (Kuntin 2018) led to correct waste segregation, allowing them to partner with a laboratory supplier to recycle centrifuge tubes, pipette tips, and other plasticware that have not held biohazardous materials (Sterilab Services 2022).

Furthermore, some manufacturers are developing biodegradable plastics specifically for laboratory use. These plastics, under the right conditions, can break down more quickly and with fewer environmental repercussions. Equally, some manufacturers offer take-back programmes for used plastic items, ensuring proper recycling or disposal. Microbiology laboratories should segregate plastic waste and collaborate with companies that can handle lab-specific recycling challenges, such as pipette tip boxes. Which other products could follow a similar scheme?

Wherever possible, laboratories could shift from single-use to reusable items. Glassware can often replace plastic containers and be sterilised for repeated use. Metal instruments can replace some plastic tools and last much longer. This is not new to microbiology laboratories where single-use plastics have often replaced more sustainable options, for example, glass Petri dishes, metal inoculating rods, and glass L-shaped spreaders, to name a few.

We can also develop protocols where microcentrifuge tubes are changed and disposed of after a few centrifugation cycles (e.g. DNA extraction); would it not be reasonable to reuse autoclaved tubes or to use biodegradable plastics? Indeed, protocols for reusing plastic consumables in routine microbiology assays already exist. For example, Soltani and colleagues (Soltani et al. 2019) showed that reusing pipette tips and tubes after chemical washing with sodium hypochlorite had no impact on PCR amplicons' quality and purity. Similarly, reusing pipette tips washed with sodium hypochlorite in automated clinical microbiology protocols for SARS-CoV-2 RT-qPCR increased efficiency, mitigated consumable shortages, and reduced costs and plastic waste (Berger et al. 2024). It is also important to consider that around the world these practices are conducted on a daily basis. Perhaps more due to economic constraints than environmental reasons, nonetheless, it is feasible. Would this be a practice that laboratories in more economically developed countries would consider adopting?

While the transition away from single-use plastics is not without challenges, particularly regarding sterility and contamination, the environmental benefits are significant. By adopting alternative materials, recycling programmes, and a culture of sustainability, microbiology laboratories can reduce the ecological footprint of scientific research. For example, Alves and colleagues (Alves et al. 2021) at the University of Edinburgh assessed and addressed the plastic use and waste of their multidisciplinary microbiology, molecular biology, and immunology laboratory facilities. Their work lead to an important reduction of single-use plastic waste and biohazard waste needing autoclaving and or incineration, thus achieving cost savings for the research institute. This case study could be used as a benchmark for other groups.

### Mindful automation

With the advancement of automation, microbiology labs in universities, industries, and the public sector are increasing their energy consumption, especially with equipment recommended to stay “on” regardless of usage. Equally, automated systems often produce more plastic waste due to high throughput processes and proprietary consumables. For example, a liquid handler from one company might not operate unless specific pipette tips are used. There are microbiology laboratories where multiple brands' liquid handlers require different pipette tips, increasing plastic waste and packaging.

Just like for most manual pipettes, where pipette tips can be bought from either the pipette manufacturer or from a generic one, automated liquid handlers offer the opportunity for a conversation across manufacturers to produce leap-frog products that can be used across technologies, or for a consumables manufacturer to come up with size-fit all alternatives.

It is worth noting that such a change would probably require a push by the consumer, it is therefore crucial that microbiologists, molecular biologists, and scientists in general request and are part of those conversations.

Achieving changes in energy management and single-use consumables would boost the benefits that automated systems already provide. For instance, the reduction of time and effort required to conduct molecular laboratory assays and the accuracy of reagent usage that often minimises the generation of chemical waste.

### Procurement

By purchasing supplies in bulk, labs can reduce the amount of packaging waste. Choosing suppliers and products that prioritise sustainability is essential. This means selecting products with minimal packaging, opting for goods made from recycled materials, selecting products which offer recycling schemes, and partnering with companies that have a clear commitment to environmental responsibility.

### Investment in research

Supporting research into new materials and methods that reduce reliance on single-use plastics, fossil fuels, and high energy operations can contribute to long-term solutions. Grants and funding opportunities specifically targeted at sustainability in the lab can incentivise innovation in this area. For instance, testing the effect of relatively higher temperatures on freezers for long term sample storage; assessing how many times pipette tips and microcentrifuge tubes could be possibly re-used, if any, for a given application; use of biodegradable consumables for growing/storing/maintaining live cultures; development of biodegradable plastics for lab applications, etc.

### Education and awareness

Perhaps the most crucial factor in driving sustainability is fostering a culture of environmental awareness within the lab. Regular training and discussions about sustainable practices encourage lab personnel to be mindful of their environmental impact. By understanding the importance of each action, from recycling a plastic tube to properly shutting down equipment, lab staff can collectively contribute to more sustainable operations.

## Potential partners

### Institutional support

The discussion above highlight the benefits of collaboration not only within a laboratory group, but also with a larger spectrum of potential partners. Clearly, universities and other institutions where microbiology laboratories are hosted would be the first avenue for a larger improvement.

Nowadays, universities get ranked on their sustainability credentials. For example, the Impact Rankings from the Times Higher Education evaluate universities performance in accordance to the UN Sustainability Goals. Similarly, the QS sustainability University Rankings measure around 1400 universities ability to tackle global environmental and social challenges. Furthermore, nation specific tables, such as the People and Planet University League in the United Kingdom, do a specific ranking of their country's higher education institutions commitment to environmental, social, and governance sustainability. It is therefore reasonable to expect and actively ask for institutional support to achieve sustainable laboratory operations.

Such support should include conducting energy and water assessments at laboratory and building scales, while conducting the necessary adjustments; evaluate appropriate insulation and ventilation installations and guiding and training researchers in their sustainability journeys. Institutions can also assist if not drive a more environmentally sustainable procurement by ordering in bulk and compile and select suppliers with sustainability commitments. Moreover, the creation of Shared Research Resources (SRR) at institutional level, where instruments, reagents, and technical expertise are shared, has demonstrated to be an invaluable tool to ensure quality, reproducible science while reducing environmental impacts of biomolecular research (Meyn et al. 2022). Microbiologist can then argue that their research institutions can achieve higher research quality outputs, economic savings, and reputation gains by supporting sustainable laboratory practices.

### Microbiology societies

Another important partnership can be formed between microbiologist and learned societies. Many microbiology societies already implement sustainability practices, such as in the organisation of events. For instance, in the latest FEMS 2023 Symposium, most of the catering was vegetarian, and single use plastic cutlery was avoided. Instead, ceramic cups were available for every coffee break, and a stainless-steel bottle was provided to all attendees so that they could refill as needed from the multiple water supplies available. Another exceptional example is the Microbial Ecology and Evolution Hubs, which in January 2024 by facilitating hybrid participation, allowed in-person attendance for local researchers, while supporting full virtual participation across Europe and North America Hubs. Hybrid conferences where travel can be flexible not only cut on carbon emissions (Achten et al. 2013), but are also more inclusive to audiences from less economically developed countries. However, microbiology societies could improve their sustainable event credentials, by asking for sustainability statements for the grants they provide for this matter, and by sponsoring events and or knowledge exchange activities specifically targeting sustainable labs.

Another key activity that microbiology societies could facilitate due to their extensive networks, is the organisation of round tables where microbiologists, institutes, industries (e.g. manufacturers, suppliers), and even policy makers could discuss alternatives and solution to environmental issues caused by research laboratories.

Also, society journals could promote scientific research in sustainability, such as this FEMS special issue on “Microbiology for a Sustainable Future”, also accepting case reports on sustainable laboratory best practices. Another example in this regard is the Applied Microbiology International new bespoke journal Sustainable Microbiology, which requires that any manuscript submission states how that piece of research addresses the UN Global Sustainability Goals. Would a similar requirement about sustainability measures/considerations taken for conducting any and every piece of research make sense? This would at least promote a thought process by authors and readers about the environmental impact of a given investigation.

## Early-career researchers: the next generation of environmental sustainability practitioners

Driven by a similar motivation to that of the present work, a group of ECR researchers worked with the publisher eLife Sciences Publications to launch the #LabWasteDay campaign on Twitter in 2019 (Howes 2019), highlighting the single-use plastic used by scientists globally. Plastic waste and other realities of the environmental impact that life-sciences research, including microbiology, has, have been recounted in numerous perspectives, case reports, and letters to the editor mostly written by ECR. On their reports, ECR narrate the contrasting perceptions that the need of sterility and sustainability entail. Some of these researchers have tried to influence change in their laboratories, with more or less success. The success stories by Alves et al. (2021) at the University of Edinburgh to cut plastic waste, as well as that of David Kuntin at the University of York (Kuntin 2018) to create a plastics decontamination station, were driven by ECR.

However, the reality of fixed-term contracts and the pressure to generate quality results worthy of publication in a short period of time, could hinder the initial enthusiasm of ECR to abide by sustainability rules. Thus, highlighting how crucial of the role of senior microbiologist is to successfully implement these changes in their laboratories.

Principal investigators, laboratory managers, and team leaders are the person of reference in every laboratory. Just as they establish the research culture and how a laboratory operates, senior scientists along with permanent technical staff should set the standards for sustainability practices in laboratory procedures. It is their prerogative, and we might argue, their responsibility, to foster a culture open to innovation committed to conducting reproducible and quality science while procuring sustainable research practices. Furthermore, permanent staff should ensure the longevity of environmentally friendly initiatives, perhaps spearheaded by ECR, beyond fix-term projects.

It is therefore paramount to bring faculty, ECR, technical staff, and students together to develop, engage, and support such changes. Having an open mindset to new practices that do not compromise on research quality and reduce the impact of research on the environment should no longer be a choice but a mandate.

In a world moved mostly by economic considerations, it is important to remember that sustainable practices not only reduce environmental footprints but also often lead to cost savings and efficiency improvements. Microbiologists, particularly environmental microbiologists, should lead by example in promoting sustainability in laboratory environments. Let's clean our own house first.
